# Occurrence and prognostic significance of cytogenetic evolution in patients with multiple myeloma

**DOI:** 10.1038/bcj.2016.15

**Published:** 2016-03-11

**Authors:** M Binder, S V Rajkumar, R P Ketterling, A Dispenzieri, M Q Lacy, M A Gertz, F K Buadi, S R Hayman, Y L Hwa, S R Zeldenrust, J A Lust, S J Russell, N Leung, P Kapoor, R S Go, W I Gonsalves, R A Kyle, S K Kumar

**Affiliations:** 1 Department of Internal Medicine, Mayo Clinic, Rochester, MN, USA; 2 Division of Hematology, Mayo Clinic, Rochester, MN, USA; 3 Division of Cytogenetics, Department of Laboratory Medicine and Pathology, Mayo Clinic, Rochester, MN, USA; 4 Division of Nephrology and Hypertension, Mayo Clinic, Rochester, MN, USA

## Abstract

Cytogenetic evaluation at the time of diagnosis is essential for risk stratification in multiple myeloma, however little is known about the occurrence and prognostic significance of cytogenetic evolution during follow-up. We studied 989 patients with multiple myeloma, including 304 patients with at least two cytogenetic evaluations. Multivariable-adjusted regression models were used to assess the associations between the parameters of interest and cytogenetic evolution as well as overall survival. The prognostic significance of baseline cytogenetic abnormalities was most pronounced at the time of diagnosis and attenuated over time. In the patients with serial cytogenetic evaluations, the presence of *t*(11;14) at the time of diagnosis was associated with decreased odds of cytogenetic evolution during follow-up (odds ratio (OR)=0.22, 95% confidence interval (CI)=0.09–0.56, *P*=0.001), while the presence of at least one trisomy or tetrasomy was associated with increased odds (OR=2.96, 95% CI=1.37–6.42, *P*=0.006). The development of additional abnormalities during the 3 years following diagnosis was associated with increased subsequent mortality (hazard ratio=3.31, 95% CI=1.73–6.30, *P<*0.001). These findings emphasize the importance of the underlying clonal disease process for risk assessment and suggest that selected patients may benefit from repeated risk stratification.

## Introduction

Two distinct oncogenic pathways have been implicated in the molecular pathogenesis of multiple myeloma.^1^ One pathway is characterized by the occurrence of translocations involving the immunoglobulin heavy chain locus (non-hyperdiploid pathway),^2^ the other one by multiple trisomies of odd-numbered chromosomes (hyperdiploid pathway).^3^ The involved pathway and the presence of specific cytogenetic abnormalities at the time of diagnosis have been shown to be of prognostic significance.^4, 5, 6, 7^ The occurrence of cytogenetic abnormalities has been implicated in disease progression^8, 9^ but it is hitherto unknown which factors are determining the subsequent development of cytogenetic abnormalities and whether or not these abnormalities are of prognostic significance later on in the course of disease. Although there is evolving consensus to reevaluate for cytogenetic high-risk features during follow-up, data regarding the acquisition, persistence and regression of other cytogenetic features are lacking.^10^ We undertook this study to identify factors associated with the subsequent evolution of cytogenetic abnormalities and to assess their prognostic significance during follow-up.

## Patients and methods

### Study population

The patients who were diagnosed with multiple myeloma at Mayo Clinic Rochester between January 2004 and December 2012 were identified by retrospective chart review. The patients with a cytogenetic evaluation via fluorescence *in situ* hybridization within 6 months of diagnosis were included in the study. Those who underwent at least two fluorescence *in situ* hybridization evaluations, including the diagnostic specimen, were included in the longitudinal subgroup.

### Cytogenetic evaluation

The bone marrow aspirates were evaluated for deletions, monosomies, trisomies, tetrasomies and translocations using locus-specific or centromere-specific fluorescence *in situ* hybridization probes. The immunoglobulin heavy chain rearrangements were evaluated using an immunoglobulin heavy chain break-apart probe and up to five potential partners (FGFR3, CCND1, CCND3, MAF and MAFB). The specimens that failed quality control and were deemed inappropriate for evaluation by the hematopathologist were excluded. The data on *t*(11;14), *t*(4;14), *t*(14;16), monosomies (9, 11, 13, 14, 15, 16, 17), trisomies (11, 13, 14, 15, 16, 17), tetrasomies (3, 7, 9, 11, 13, 15, 17), del(13q), and del(17p) were obtained for all the specimens. The data on *t*(6;14) and *t*(14;20) were obtained from 2009 onward and the analyses were adjusted accordingly. Cytogenetic evolution was defined as a new deletion, monosomy, trisomy, tetrasomy or translocation during follow-up. The presence of del(17p), *t*(14;16) or *t*(14;20) was considered a high-risk abnormality. Hyperdiploidy was defined as the presence of multiple (⩾2) trisomies.

### Statistical analysis

Multivariable-adjusted logistic regression models were used to assess the associations between the parameters of interest at diagnosis and the presence of cytogenetic evolution in the follow-up specimens. All the models were adjusted for sex, age, the presence of high-risk abnormalities, the number of abnormalities at the time of diagnosis and the time between the first and last cytogenetic evaluation. Overall survival estimates were calculated using the method described by Kaplan and Meier.^11^ The log-rank test was used to assess the differences in survival distributions. Multivariable-adjusted Cox proportional hazards models^12^ were used to assess the effect of cytogenetic evolution on overall survival. All the models were adjusted for sex, age at diagnosis and the number of cytogenetic evaluations. The model assessing the prognostic significance of additional cytogenetic abnormalities during the 3 years after diagnosis was additionally adjusted for the presence of high-risk abnormalities and the number of abnormalities at diagnosis. Likelihood ratio tests were used to assess the goodness of fit of nested models. The *χ*^2^ or Fisher's exact test was used to assess the distribution of cytogenetic abnormalities in the subgroups.

## Results

Between January 2004 and December 2012, there were 989 patients with a new diagnosis of multiple myeloma with cytogenetic data at the Mayo Clinic Rochester. Three hundred and four patients (31%) underwent at least one additional cytogenetic evaluation during follow-up either at the time of disease progression (98%) or during the evaluation for autologous hematopoietic stem cell transplantation (2%). The median time between the first and last cytogenetic evaluation was 14 months (1–88). The patients who underwent serial cytogenetic evaluations were younger at the time of diagnosis and experienced longer overall survival compared with the patient with a single cytogenetic evaluation (median 6.7 versus 5.1 years, *P<*0.001), reflecting the fact that these patients had to survive long enough to undergo repeated cytogenetic evaluation. The distribution of cytogenetic features in the two subgroups was very similar. The patient characteristics, cytogenetic features at diagnosis and survival experience of the entire cohort as well as the two subgroups are summarized in Table 1.

### Prognostic significance of baseline cytogenetic features during follow-up

**Figure 1:**
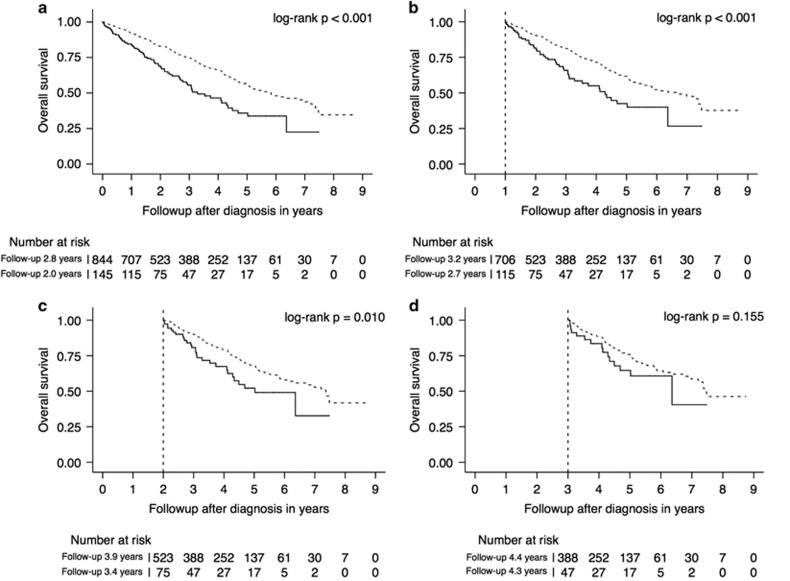
Kaplan–Meier overall survival estimates stratified by the presence of cytogenetic high-risk features at the time of diagnosis (solid line): Landmark analysis with patients entering the cohort at the time of diagnosis (**a**), and the survivors 1 (**b**), 2 (**c**) and 3 (**d**) years after diagnosis.

**Figure 2:**
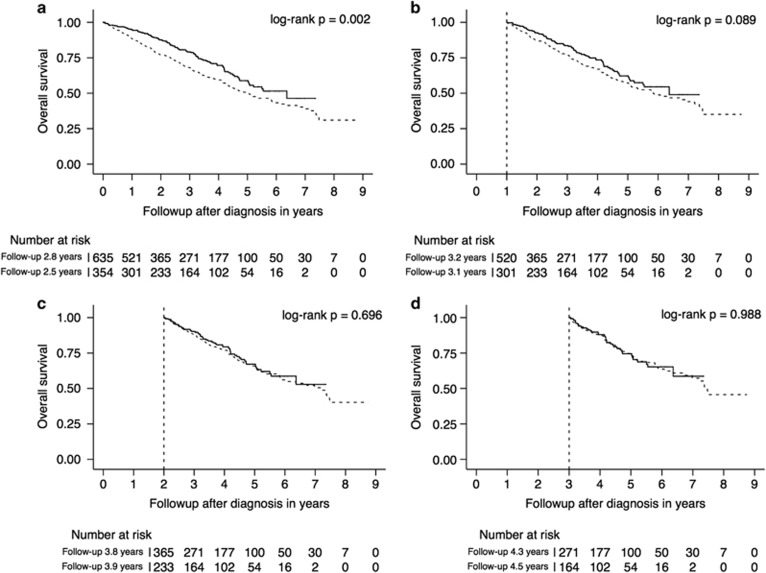
Kaplan–Meier overall survival estimates stratified by the presence of a hyperdiploid clone at the time of diagnosis (solid line): Landmark analysis with patients entering the cohort at the time of diagnosis (**a**), and the survivors 1 (**b**), 2 (**c**) and 3 (**d**) years after diagnosis.

Consistent with prior reports,^3, 6, 7^ the presence of cytogenetic high-risk features and the absence of a hyperdiploid clone at diagnosis were associated with shorter overall survival (Table 1). However, the effects of these prognostic factors were attenuated over time. The presence of high-risk features was no longer associated with overall survival in those who survived 3 years, the absence of a hyperdiploid clone was no longer of prognostic significance in those who survived 1 year after diagnosis. Figures 1 and 2 show the Kaplan–Meier overall survival estimates for the entire cohort and those patients who survived 1, 2 and 3 years after diagnosis, stratified by the aforementioned prognostic factors. The corresponding multivariable-adjusted hazard ratios are shown in Table 2.

### Occurrence of cytogenetic evolution

The patients with a hyperdiploid clone at diagnosis were more frequently found to develop new cytogenetic abnormalities later on during the course of disease, especially the acquisition of additional copies of chromosomes was more common (Table 3). Although the propensity to develop new abnormalities was different, the types of new abnormalities were very similar in the two groups: Monosomy 13, trisomy 11, tetrasomy 15 and deletion 17p were the most common new abnormalities in both the groups. The presence of additional copies of chromosomes (any trisomy or tetrasomy at diagnosis) was associated with increased odds of new abnormalities during follow-up (OR (odds ratio)=2.96, 95% CI (95% confidence interval)=1.37–6.42, *P*=0.006) while the presence of *t*(11;14) was associated with decreased odds (OR=0.22, 95% CI=0.09–0.56, *P*=0.001), adjusting for sex, age, the presence of high-risk abnormalities, the number of abnormalities at the time of diagnosis and the time between first and last cytogenetic evaluation. The presence of high-risk abnormalities at the time of diagnosis was not associated with increased odds of new abnormalities during follow-up (*P*=0.267). In addition, adjusting for autologous hematopoietic stem cell transplantation status did not significantly change the parameter estimates or improve model fit (*P*=0.427). The other two translocations with sufficient data to analyze, *t*(4;14) and *t*(14;16), were not associated with the development of new abnormalities during the follow-up (*P*=0.219 and *P*=0.624, respectively).

During the follow-up, 145 patients (47.7%) were found to have lost at least one abnormality (median 1, range 1–8) that was present on prior cytogenetic evaluations. The most common lost abnormalities were monosomy 13, trisomy 15, tetrasomy 11 and deletion 17p. Adjusting for the same factors as before, the number of abnormalities at the time of diagnosis was the only characteristic associated with increased odds of loss of abnormalities during the follow-up (OR=1.49 for each abnormality present at the time of diagnosis, 95% CI=1.20–1.85, *P<*0.001). Both the development of new (*n*=1) and the loss of existing translocations (*n*=3) was rare.

### Prognostic significance of cytogenetic evolution

**Figure 3:**
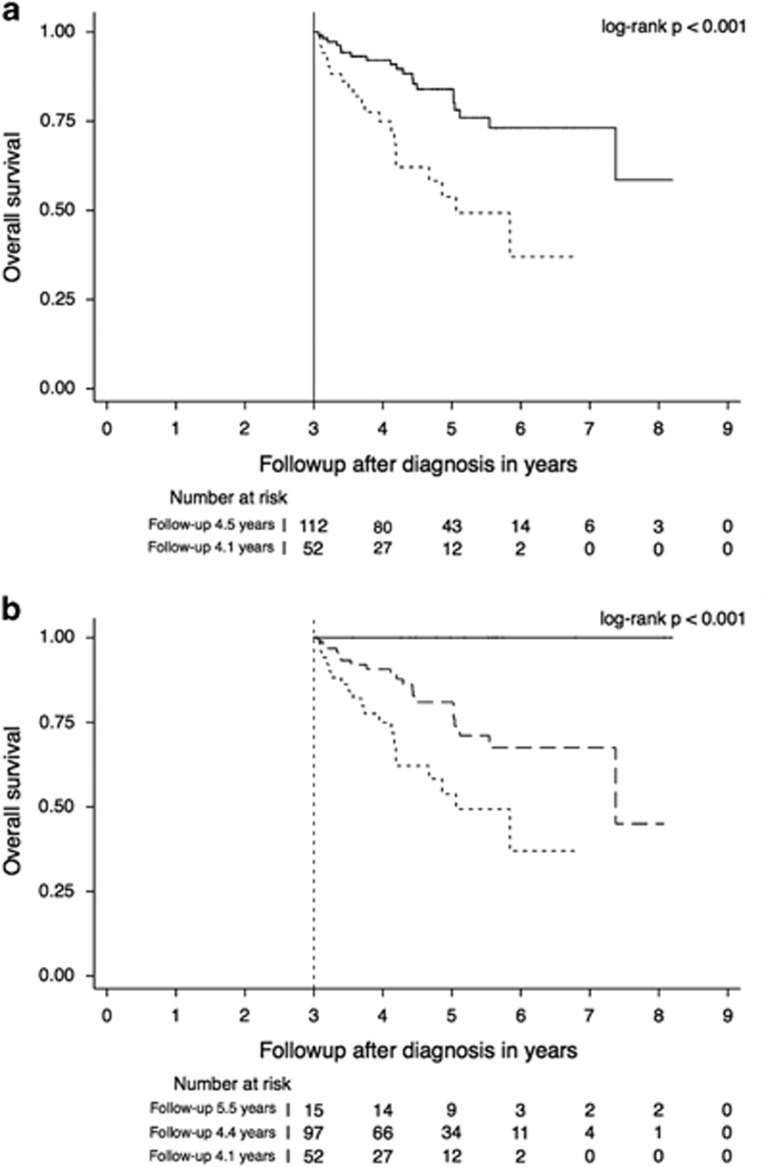
(**a**) Kaplan–Meier overall survival estimates stratified by cytogenetic stability (solid line) versus new cytogenetic abnormalities (dashed line), 3 years after diagnosis in the 164 patients who survived at least 3 years (landmark analysis). (**b**) Kaplan–Meier overall survival estimates further stratified by cytogenetic normalization (solid line) versus cytogenetic stability (long dashed line) versus new cytogenetic abnormalities (short dashed line).

One hundred and sixty four of the 304 patients with serial cytogenetic evaluations were alive 3 years after diagnosis. Figure 3a shows the Kaplan–Meier overall survival estimates for these 164 patients, stratified by the development of new cytogenetic abnormalities. Development of new cytogenetic abnormalities during the 3 years after diagnosis was associated with increased subsequent mortality (hazard ratio=3.31, 95% CI=1.73–6.30, *P<*0.001), adjusting for sex, age, the presence of high-risk abnormalities and the number of abnormalities at the time of diagnosis. In addition, adjusting for the time between the first and last cytogenetic evaluation and autologous hematopoietic stem cell transplantation status did not significantly change the parameter estimates or improve model fit (*P*=0.114 and *P*=0.360, respectively).

Figure 3b further stratifies the group of patients without new abnormalities into those who had abnormalities at the time of diagnosis but not during follow-up (normalization) and those who had stable abnormalities over time (stability). Both the patients with stable abnormalities and the patients with new abnormalities experienced worse overall survival than the patients with normalization (*P*=0.026 and *P*=0.002, respectively). As there were no deaths observed in the normalization group, no hazard ratios were estimated.

The proportion of patients with cytogenetic high-risk features at diagnosis was identical in those who developed additional abnormalities later on and those who did not (14% in both groups, *P*=1.000). The subsequent overall survival experience of the 10 patients who developed high-risk features during the follow-up was not statistically different from the 42 patients developing other cytogenetic abnormalities (*P*=0.392). Likewise, the subsequent overall survival experience of the 36 patients who developed additional copies of chromosomes was not statistically different from the 16 patients developing other cytogenetic abnormalities (*P*=0.217).

## Discussion

Over the last decade several new treatment options including immunomodulators, proteasome-inhibitors and more recently a histone deacetylase inhibitor and a monoclonal antibody^13^ have become available for patients with multiple myeloma. Immunotherapies including monoclonal antibodies^14^ and transduction of autologous T cells to target specific surface antigens^15^ are currently being investigated. The use of novel agents and risk-adapted treatment strategies have led to an increase in response rates and overall survival in patients with newly diagnosed multiple myeloma and patients with advanced disease.^16, 17, 18, 19, 20, 21, 22, 23^ Although the demographic characteristics of patients treated at Mayo Clinic Rochester have changed little over the past 30 years, the median overall survival has almost doubled (median overall survival 5.3 years in the current cohort versus 2.8 years in the 1027 patients diagnosed between 1985 and 1998) and more than half of the patients with unfavorable cytogenetics were alive 3 years after diagnosis.^24^ Risk-adapted treatment strategies currently solely rely on cytogenetic evaluation at the time of diagnosis,^25^ and to our knowledge, no prior studies exist to examine the impact of cytogenetic evolution throughout the course of disease.

Our data suggest that the presence of high-risk abnormalities or a hyperdiploid clone at the time of diagnosis is most informative for the time immediately following diagnosis and that these effects are attenuated over time. With every year, a patient surviving the impact of these cytogenetic findings becomes less pronounced. Both treatment exerting selective pressure on the different clones present at diagnosis and natural progression of the disease are plausible explanations for these observed alterations of the underlying disease process. Clones harboring the cytogenetic abnormalities detected at the time of diagnosis may no longer be present or driving the disease process 1, 2 or 3 years later. This seemed to be relevant for the small subgroup of patients with a normalization of their cytogenetic profile during the 3 years following diagnosis that experienced excellent subsequent overall survival. Although we did not investigate treatment effects on cytogenetic evolution, regression towards the mean is likely contributing to the association observed between the number of abnormalities at the time of diagnosis and the loss of these abnormalities during follow-up. Future studies are needed to investigate the impact of specific treatment regimens on cytogenetic evolution over time.

Although the newly evolved abnormalities were similar between patients with and without a hyperdiploid clone at diagnosis, the propensity to develop these abnormalities was different. Among the examined translocations, *t*(11;14) was associated with increased cytogenetic stability during the follow-up. This translocation upregulates cyclin D1 and is less commonly seen in hyperdiploid disease. It has been associated with lymphoplasmacytic morphology and lower serum monoclonal protein concentrations in a prior study but the greater median survival (49.6 versus 38.7 months) did not reach statistical significance.^26^ Its presence was associated with better 1-year overall survival in a small cohort of patients with primary plasma cell leukemia.^27^ These findings suggesting more favorable outcomes may be a marker of increased cytogenetic stability in this patient population.

In our cohort, cytogenetic stability was of prognostic significance later on in the course of disease. Those patients who survived 3 years after diagnosis without the development of new cytogenetic abnormalities experienced increased subsequent overall survival compared with those who developed new abnormalities. Although there is consensus to reassess the presence of high-risk abnormalities during follow-up,^10^ the difference in subsequent overall survival was not driven by a differential distribution of high-risk abnormalities in our cohort. In majority of the cases, it was rather the acquisition of additional copies of chromosomes, potentially reflecting an underlying active or genetically unstable clone driving the disease process at that point. With the upcoming availability of new agents, further improvement in survival is to be expected in the near future. Today, the vast majority of patients with multiple myeloma are experiencing overall survival beyond 3 years after diagnosis. Our data suggest that the cytogenetic risk strata established at the time of diagnosis become less and less informative as the disease progresses. The underlying clonal process seems to evolve over time and so do associated prognostic factors. An assessment of cytogenetic stability proved to be relevant for those patients who survived 3 years or more after diagnosis. The distinct cytogenetic features present at the time of diagnosis were associated with cytogenetic stability during the follow-up. These findings suggest that selected subgroups of patients may benefit from repeated risk stratification during the follow-up.

The ability to draw firm conclusions from these data is limited by the retrospective nature of this study. The associations remained stable after adjusting for potential confounding factors including patient and disease characteristics, suggesting an independent predictive or prognostic value of these cytogenetic features, respectively. Careful assessment of patient and disease characteristics, adjustment for known confounding factors and sensitivity analyses yielded stable effect estimates. However, the presence of residual confounding accounting for parts of the observed associations cannot be completely excluded. The strengths of this study include a large single-center cohort of patients evaluated and treated in a uniform manner, relative completeness of data and several years of follow-up.

## Figures and Tables

**Table 1:** Characteristics of the whole cohort of 989 patients with multiple myeloma (stratified by the number of cytogenetic evaluations)

	*Entire cohort*	*Serial FISH*	*Single FISH*
Men (*n* (%))	585 (59)	192 (63)	393 (57)
Age at diagnosis (years)	63 (22–95)	61 (32–82)	65 (22–95)
Follow-up (years)	2.7 (0–9)	3.7 (0–8)	2.0 (0–9)
Overall survival (years)	5.3 (4.8–6.4)	6.7 (5.0–7.4)	5.1 (4.3–6.1)
			
*Cytogenetic features at diagnosis (*n *(%))*			
Standard or intermediate risk	844 (85)	253 (83)	591 (86)
High risk	145 (15)	51 (17)	94 (14)
			
*Overall survival by cytogenetic features (years (95% CI))*			
Standard or intermediate risk	5.8 (5.1–7.2)	7.0 (5.1–NR)	5.3 (4.5–6.4)
High risk	3.3 (2.7–4.3)	4.1 (3.1–NR)	2.4 (1.8–4.7)
			
*Karyotype at diagnosis (*n *(%))*			
Hyperdiploid karyotype	354 (36)	112 (37)	243 (35)
Non-hyperdiploid karyotype	635 (64)	192 (63)	442 (65)
			
*Overall survival by karyotype (years (95% CI))*			
Hyperdiploid karyotype	6.4 (5.0–NR)	NR (5.0–NR)	6.4 (4.4–NR)
Non-hyperdiploid karyotype	5.0 (4.3–5.8)	5.8 (4.7–7.4)	4.6 (4.1–5.8)
			
*Cytogenetic abnormalities at diagnosis (*n *(%))*			
Translocations	337 (34)	101 (33)	236 (34)
Monosomies	400 (40)	130 (43)	270 (39)
Trisomies	541 (55)	172 (57)	369 (54)
Tetrasomies	119 (12)	33 (11)	86 (13)
Deletions	196 (20)	63 (21)	133 (19)

Abbreviations: CI, confidence interval; FISH, fluorescence *in situ* hybridization; NR, not reached.

The data are given as median (range) unless denoted otherwise. NR, not reached.

**Table 2:** Multivariable-adjusted hazard ratios (overall survival) for the presence of cytogenetic high-risk features and a hyperdiploid clone at the time of diagnosis and the three consecutive years (landmark analysis)

*Population*	*Subgroup/all patients*	*HR (95% CI)*	P-*value*
*Cytogenetic high-risk features at diagnosis*			
At diagnosis	145/989	1.90 (1.46–2.48)	<0.001
1-year survivors	115/822	1.82 (1.33–2.51)	<0.001
2-year survivors	75/598	1.71 (1.14–2.57)	0.010
3-year survivors	47/435	1.51 (0.88–2.62)	0.138
			
*Hyperdiploid clone at diagnosis*			
At diagnosis	354/989	0.67 (0.53–0.85)	0.001
1-year survivors	301/822	0.77 (0.59–1.01)	0.061
2-year survivors	233/598	0.92 (0.67–1.27)	0.603
3-year survivors	164/435	0.96 (0.64–1.44)	0.845

Abbreviations: CI, confidence interval; HR, hazard ratio.

All the models are additionally adjusted for sex, age at diagnosis and the number of cytogenetic evaluations.

**Table 3:** Cytogenetic evolution during follow-up in 304 patients with multiple myeloma stratified by FISH karyotype at the time of diagnosis

	*Hyperdiploid (*n=*112)*	*Non-hyperdiploid (*n=*192)*	P-*value*
New abnormality	60 (54%)	69 (36%)	0.003
New monosomy	6 (5%)	14 (7%)	0.634
New trisomy	37 (33%)	33 (17%)	0.002
New tetrasomy	29 (26%)	19 (10%)	<0.001
New deletion	13 (12%)	12 (6%)	0.129
New translocation	1 (1%)	0 (0%)	0.368

Abbreviation: FISH, fluorescence *in situ* hybridization.

The data are given as count (percent) unless denoted otherwise.

## References

[bib1] Smadja NV, Fruchart C, Isnard F, Louvet C, Dutel JL, Cheron N et al. Chromosomal analysis in multiple myeloma: cytogenetic evidence of two different diseases. Leukemia 1998; 12: 960–969. 9639426 10.1038/sj.leu.2401041

[bib2] Fonseca R, Debes-Marun CS, Picken EB, Dewald GW, Bryant SC, Winkler JM et al. The recurrent IgH translocations are highly associated with nonhyperdiploid variant multiple myeloma. Blood 2003; 102: 2562–2567. 12805059 10.1182/blood-2003-02-0493

[bib3] Debes-Marun CS, Dewald GW, Bryant S, Picken E, Santana-Dávila R, González-Paz N et al. Chromosome abnormalities clustering and its implications for pathogenesis and prognosis in myeloma. Leukemia 2003; 17: 427–436. 12592343 10.1038/sj.leu.2402797

[bib4] Tricot G, Barlogie B, Jagannath S, Bracy D, Mattox S, Vesole DH et al. Poor prognosis in multiple myeloma is associated only with partial or complete deletions of chromosome 13 or abnormalities involving 11q and not with other karyotype abnormalities. Blood 1995; 86: 4250–4256. 7492784

[bib5] Pérez-Simón JA, García-Sanz R, Tabernero MD, Almeida J, González M, Fernández-Calvo J et al. Prognostic value of numerical chromosome aberrations in multiple myeloma: a FISH analysis of 15 different chromosomes. Blood 1998; 91: 3366–3371. 9558394

[bib6] Smadja NV, Bastard C, Brigaudeau C, Leroux D, Fruchart C, Groupe Français de Cytogénétique Hématologique. Hypodiploidy is a major prognostic factor in multiple myeloma. Blood 2001; 98: 2229–2238. 11568011 10.1182/blood.v98.7.2229

[bib7] Avet-Loiseau H, Attal M, Moreau P, Charbonnel C, Garban F, Hulin C et al. Genetic abnormalities and survival in multiple myeloma: the experience of the Intergroupe Francophone du Myélome. Blood 2007; 109: 3489–3495. 17209057 10.1182/blood-2006-08-040410

[bib8] Chiecchio L, Dagrada GP, Ibrahim AH, Dachs Cabanas E, Protheroe RK, Stockley DM et al. Timing of acquisition of deletion 13 in plasma cell dyscrasias is dependent on genetic context. Haematologica 2009; 94: 1708–1713. 19996118 10.3324/haematol.2009.011064 PMC2791926

[bib9] Rajkumar SV, Gupta V, Fonseca R, Dispenzieri A, Gonsalves WI, Larson D et al. Impact of primary molecular cytogenetic abnormalities and risk of progression in smoldering multiple myeloma. Leukemia 2013; 27: 1738–1744. 23515097 10.1038/leu.2013.86 PMC3773463

[bib10] Munshi NC, Anderson KC, Bergsagel PL, Shaughnessy J, Palumbo A, Durie B et al. Consensus recommendations for risk stratification in multiple myeloma: report of the International Myeloma Workshop Consensus Panel 2. Blood 2011; 117: 4696–4700. 21292777 10.1182/blood-2010-10-300970 PMC3293763

[bib11] Kaplan EL, Meier P. Nonparametric estimation from incomplete observations. J Am Stat Assoc 1958; 53: 457.

[bib12] Cox DR. Regression models and life-tables. J R Stat Soc B 1972; 34: 187–220.

[bib13] Lokhorst HM, Plesner T, Laubach JP, Nahi H, Gimsing P, Hansson M et al. Targeting CD38 with daratumumab monotherapy in multiple myeloma. N Engl J Med 2015; 373: 1207–1219. 26308596 10.1056/NEJMoa1506348

[bib14] Lonial S, Dimopoulos M, Palumbo A, White D, Grosicki S, Spicka I et al. Elotuzumab therapy for relapsed or refractory multiple myeloma. N Engl J Med 2015; 373: 621–631. 26035255 10.1056/NEJMoa1505654

[bib15] Garfall AL, Maus MV, Hwang WT, Lacey SF, Mahnke YD, Melenhorst JJ et al. Chimeric antigen receptor T cells against CD19 for multiple myeloma. N Engl J Med 2015; 373: 1040–1047. 26352815 10.1056/NEJMoa1504542 PMC4646711

[bib16] Rajkumar SV, Hayman SR, Lacy MQ, Dispenzieri A, Geyer SM, Kabat B et al. Combination therapy with lenalidomide plus dexamethasone (Rev/Dex) for newly diagnosed myeloma. Blood 2005; 106: 4050–4053. 16118317 10.1182/blood-2005-07-2817 PMC1895238

[bib17] Rajkumar SV, Blood E, Vesole D, Fonseca R, Greipp PR, Eastern Cooperative Oncology Group. Phase III clinical trial of thalidomide plus dexamethasone compared with dexamethasone alone in newly diagnosed multiple myeloma: a clinical trial coordinated by the Eastern Cooperative Oncology Group. J Clin Oncol 2006; 24: 431–436. 16365178 10.1200/JCO.2005.03.0221

[bib18] Rajkumar SV, Rosiñol L, Hussein M, Catalano J, Jedrzejczak W, Lucy L et al. Multicenter, randomized, double-blind, placebo-controlled study of thalidomide plus dexamethasone compared with dexamethasone as initial therapy for newly diagnosed multiple myeloma. J Clin Oncol 2008; 26: 2171–2177. 18362366 10.1200/JCO.2007.14.1853 PMC3904367

[bib19] Kumar SK, Rajkumar SV, Dispenzieri A, Lacy MQ, Hayman SR, Buadi FK et al. Improved survival in multiple myeloma and the impact of novel therapies. Blood 2008; 111: 2516–2520. 17975015 10.1182/blood-2007-10-116129 PMC2254544

[bib20] Rajkumar SV, Jacobus S, Callander NS, Fonseca R, Vesole DH, Williams ME et al. Lenalidomide plus high-dose dexamethasone versus lenalidomide plus low-dose dexamethasone as initial therapy for newly diagnosed multiple myeloma: an open-label randomised controlled trial. Lancet Oncol 2010; 11: 29–37. 19853510 10.1016/S1470-2045(09)70284-0 PMC3042271

[bib21] Richardson PG, Weller E, Lonial S, Jakubowiak AJ, Jagannath S, Raje NS et al. Lenalidomide, bortezomib, and dexamethasone combination therapy in patients with newly diagnosed multiple myeloma. Blood 2010; 116: 679–686. 20385792 10.1182/blood-2010-02-268862 PMC3324254

[bib22] Zonder JA, Crowley J, Hussein MA, Bolejack V, Moore DF, Whittenberger BF et al. Lenalidomide and high-dose dexamethasone compared with dexamethasone as initial therapy for multiple myeloma: a randomized Southwest Oncology Group trial (S0232). Blood 2010; 116: 5838–5841. 20876454 10.1182/blood-2010-08-303487 PMC3031379

[bib23] Sonneveld P, Asselbergs E, Zweegman S, van der Holt B, Kersten MJ, Vellenga E et al. Phase 2 study of carfilzomib, thalidomide and dexamethasone as induction/consolidation therapy for newly diagnosed multiple myeloma. Blood 2014; 125: 449–456. 25398935 10.1182/blood-2014-05-576256 PMC4300390

[bib24] Kyle RA, Gertz MA, Witzig TE, Lust JA, Lacy MQ, Dispenzieri A et al. Review of 1027 patients with newly diagnosed multiple myeloma. Mayo Clin Proc 2003; 78: 21–33. 12528874 10.4065/78.1.21

[bib25] Palumbo A, Avet-Loiseau H, Oliva S, Lokhorst HM, Goldschmidt H, Rosinol L et al. Revised international staging system for multiple myeloma: a report from International Myeloma Working Group. J Clin Oncol 2015; 33: 2863–2869. 26240224 10.1200/JCO.2015.61.2267 PMC4846284

[bib26] Fonseca R, Blood EA, Oken MM, Kyle RA, Dewald GW, Bailey RJ et al. Myeloma and the t(11;14)(q13;q32); evidence for a biologically defined unique subset of patients. Blood 2002; 99: 3735–3741. 11986230 10.1182/blood.v99.10.3735

[bib27] Avet-Loiseau H, Daviet A, Brigaudeau C, Callet-Bauchu E, Terré C, Lafage-Pochitaloff M et al. Cytogenetic, interphase, and multicolor fluorescence *in situ* hybridization analyses in primary plasma cell leukemia: a study of 40 patients at diagnosis, on behalf of the Intergroupe Francophone du Myélome and the Groupe Français de Cytogénétique Hématologique. Blood 2001; 97: 822–825. 11157506 10.1182/blood.v97.3.822

